# Age-related M1/M2 phenotype changes in circulating monocytes from healthy/unhealthy individuals

**DOI:** 10.18632/aging.101465

**Published:** 2018-06-08

**Authors:** Andrea Costantini, Nadia Viola, Antonella Berretta, Roberta Galeazzi, Giulia Matacchione, Jacopo Sabbatinelli, Gianluca Storci, Serena De Matteis, Luca Butini, Maria Rita Rippo, Antonio Domenico Procopio, Daniele Caraceni, Roberto Antonicelli, Fabiola Olivieri, Massimiliano Bonafè

**Affiliations:** 1 Department of Clinical and Molecular Sciences, DISCLIMO, Università Politecnica delle Marche, Ancona, Italy; 2 Azienda Ospedaliero Universitaria Ospedali Riuniti, Ancona, Italy; 3 Clinical and Molecular Laboratory, I.N.R.C.A. (Italian National Research Centre on Aging)-IRCCS, Ancona, Italy; 4 Department of Experimental, Diagnostic and Specialty Medicine, DIMES, Alma Mater Studiorum, Bologna, Italy; 5 Biosciences Laboratory, Istituto Scientifico Romagnolo per lo Studio e la Cura dei Tumori (IRST) IRCCS, Meldola, Italy; 6 Center of Clinical Pathology and Innovative Therapy, I.N.R.C.A. (Italian National Research Centre on Aging)-IRCCS, Ancona, Italy;; 7 Department of Cardiology, I.N.R.C.A. (Italian National Research Centre on Aging)-IRCCS, Ancona, Italy; * Equal contribution

**Keywords:** aging, M1/M2 monocytes, NK/NK-T cells, acute myocardial infarction

## Abstract

Macrophage polarization is a candidate biomarker of disease-related inflammatory status, but its modulation during aging has not been investigated. To do this, the M1/M2 profile was assessed by CD80/CD163 gating in classical (CD14^++^CD16^-^), intermediate (CD14^++^CD16^+^), and non-classical (CD14^low^CD16^+^) monocytes from 31 healthy subjects (CTRs) of different ages. Cytofluorimetric analysis showed a significantly different CD80/CD163 distribution in the three subsets, as more than 80% of classical and intermediate monocytes were CD80^+^CD163^+^, whereas most non-classical monocytes were CD80^-^CD163^-^ and CD163^+^. Non-classical CD163^+^ monocytes were significantly higher whereas classical CD163^+^ and CD80^-^CD163^-^ monocytes significantly lower in older than younger CTRs (cut-off, 65 years), suggesting different age-related trends for M2 subsets. To establish whether an M1/M2 imbalance could be associated with disease, 21 patients with acute myocardial infarction (AMI) were compared with older CTRs. The AMI patients showed a significantly decreased proportion of CD163^+^CD80^+^ and an increased proportion of CD163^+^ and CD163^-^CD80^-^ cells among classical monocytes, opposite trends to those observed in healthy aging. Moreover, a significantly greater proportion of intermediate and non-classical CD80^+^ monocytes suggested a shift to a pro-inflammatory phenotype. Overall, CD163/CD80 cytofluorimetric characterization of circulating monocytes provides additional information about their polarization and could be an innovative tool to monitor aging.

## Introduction

The low-grade, chronic inflammatory state affecting aging organisms – inflamm-aging – is among the major risk factors for the development of the most common human age-related diseases (ARDs) [1]. Increasing evidence suggests that inflamm-aging is underpinned by monocytes/macrophages, while the acquisition of a senescent phenotype – a phenomenon that has been defined as macroph-aging – impairs the ability of immune cells to cope with stressors, thus contributing to immunosenescence [2]. In this framework, the role of macrophage polarization in the modulation of inflammatory and repair processes is attracting growing interest. Monocytes are a heterogeneous and plastic population of innate myeloid cells involved in the response to damage-associated and pathogen-associated molecular patterns (respectively DAMPS and PAMPS) [3]. In humans monocytes, which account for 5–10% of peripheral leucocytes, have been classified into three subtypes based on the relative surface expression of LPS co-receptor CD14 and FCγIII receptor CD16 [4]. Classical monocytes (CD14^++^CD16^-^) represent approximately 80% of the total population, whereas monocytes expressing CD16, which account for about 20%, have been further classified into two subtypes: non-classical (CD14^++^CD16^+^) and intermediate (CD14^low^CD16^+^) monocytes [4]. Macrophage differentiation from monocytes occurs in tissues in concomitance with the acquisition of a functional phenotype depending on the local environment. The two main, and opposite activities of macrophages have led them to be classified into pro-inflammatory classically activated macrophages (M1) and anti-inflammatory and immunoregulatory alternatively activated macrophages (M2) [5]. Despite its value, this classification is however insufficient to describe the diverse phenotypes and functions of monocytes/macrophages *in vivo*. Intense research is being devoted to associate the polarization profiles, seen *in vitro* in relation to specific stimuli, with circulating and/or tissue macrophage polarization in health and disease conditions. Clearly, *in vitro* models are unable to mimic the complex environment that influences the M1/M2 balance *in vivo*, and since macrophages can develop mixed M1/M2 phenotypes, novel *in vivo* detection strategies are required. Several biomarkers have been associated with M1/M2 profiles. CD163, the high-affinity scavenger receptor for the haemoglobin-haptoglobin complex, is selectively expressed on M2 macrophages and monocytes [6], whereas CD80 (B7-1), a costimulatory signal for T cell activation and survival, is preferentially expressed on M1 macrophages. The different functions of M1 and M2 macrophages have been demonstrated in various tissues that are relevant for ARDs [7]. A significant relationship has been described between macrophage polarization and the proneness to develop atherosclerotic plaques [8–10].

Recently, M1/M2 profiles have also been examined in circulating monocytes, both in physiological and pathological conditions. Analysis of monocytes from patients affected by ARDs has led to the observation of significant associations between CD163^+^ monocytes and diabetes and its complications [11,12]. The investigation of CD163^+^ circulating M2-like monocytes as diagnostic biomarkers in breast cancer has suggested that they may have a role in reflecting cancer progression [13].

Of interest, some evidence suggested that monocytes frequency and differentiation can be influenced by activation of NK cells, and this interplay was associated with diseases outcome, *i.e.* neuroinflammation and lung inflammation [14,15].

Since data on the M1/M2 phenotype of circulating monocytes in healthy aging are not available, this study was undertaken to analyse monocyte profiles in healthy subjects of different ages using flow cytometry. To unravel possible associations between monocytes polarization and NK cells, also NT and NKT cells were analysed. Finally, to establish whether an M1/M2 imbalance could be disease-associated, the M1/M2 phenotype of elderly healthy subjects was compared with the one of elderly patients with acute myocardial infarction (AMI).

## RESULTS

### Haematological and biochemical characteristics of the study groups

Participants were 21 AMI patients and 31 healthy control (CTR) individuals. All AMI patients were older than 65 years. CTR subjects were divided into a younger and an older age group (cut-off, 65 years). The haematological and biochemical characteristics of participants are summarized in Table 1. A significant increased percentage and number (both p<0.05) of circulating monocytes was observed in older compared to younger CTR.

**Table 1:** The haematological and biochemical characteristics of participants.

**Participants**	**Younger CTR** **(Age < 65)** **(n=12)**	**Older CTR** **(Age ≥ 65)** **(n=19)**	**AMI** **(Age > 65)** **(n=21)**
Age (years)	48±8.4	83±6	85±5
Gender M-F (Number)	6-6	12-7	11-10
Glucose (mg/dL)	92.9±8.5	94.5±1.1	100.5±4.2*
Total cholesterol (mg/dL)	196.2±26.8	202.2±31.2	220.2±37.9*
White blood cells (n/mm^3^)	6800±1500	6600±2100	11,000±8400*
Monocytes (%)	6.3±1.4	7.9±1.6#	7.2±3.1
Monocytes (n/mm^3^)	416.4±64.5	526.4±179.2#	632.3±254.7
Haemoglobin (g/dL)	14.5±1.4	14.2±1.7	11±1.8*
Hs-TnT (pg/mL)	0.02±0.01	0.02±0.01	676.9±833.5*

AMI patients had higher levels of total cholesterol and fasting glucose, a higher WBC count, and significantly lower (p<0.05) Hb levels compared with older CTR.

### Flow cytometry analysis of circulating monocytes

**Figure 1:**
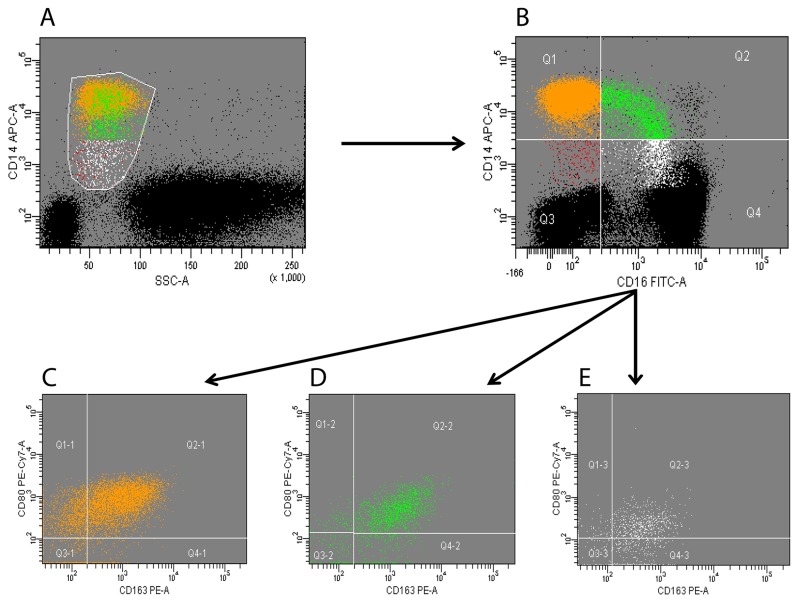
**Flow cytometry analysis of circulating monocytes.** Peripheral blood mononuclear cells (PBMCs) were collected and analyzed; monocytes were identified according to side scatter and CD14 profile (panel **A**). CD14+ cells (total monocytes) were subsequently separated according to CD16 expression into classical (orange), intermediate (green) and non-classical (white) subsets (panel **B**). Expression of CD80 and CD163 was analyzed on each of the three subsets (panels **C**-**E**). Black dots in panels **A**-**B** represent CD14-negative PBMCs (i.e. lymphocytes and granulocytes). A minimum of 200,000 PBMCs were acquired for each sample. The figure is representative of a single experiment.

Peripheral blood monocytes were identified according to side scatter and CD14 profile. CD14^+^ cells (total monocytes) were subsequently separated according to CD16 expression into in classical (CD14^++^CD16^-^), intermediate (CD14^++^CD16^+^), and non-classical (CD14^low^CD16^+^) monocytes subsets. Expression of CD80 and CD163 was finally analyzed on each of the three subset (Figure 1).

### Monocyte trends in healthy subjects

**Figure 2:**
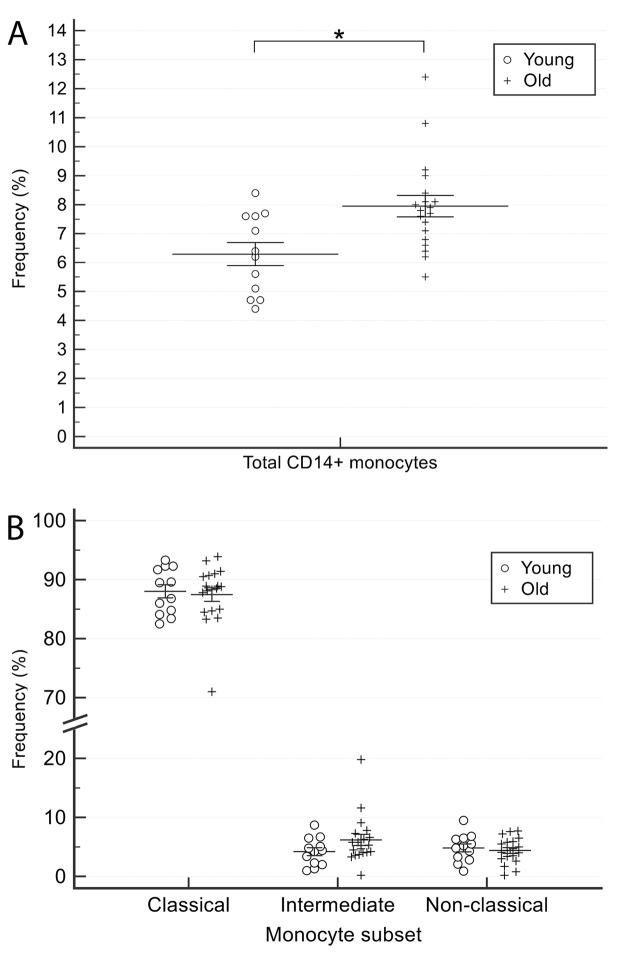
**Comparison of total monocyte (CD14^+^) and monocytes subsets (classical, intermediate and non-classical) proportions between younger and older healthy subjects.** (**A**) total monocyte (CD14+) and (**B**) monocytes subsets (classical, intermediate and non-classical). Young = 12 healthy subjects younger than 65 years. Old = 19 healthy subjects older than 65 years. Data are expressed as mean±SEM. *p<0.05.

The percentage of total (CD14^+^) monocytes was significantly (p<0.05) greater in older than in younger CTR subjects (Table 1 and Figure 2A). CD16 expression was determined to calculate the frequency of classical (CD14^++^CD16^-^), intermediate (CD14^++^CD16^+^), and non-classical (CD14^low^CD16^+^) monocyte subsets. Data analysis showed that the differences between younger and older CTR subjects for this parameter were not significant (Figure 2B). Collectively, these data suggest that healthy aging is associated with a significantly increased proportion of total monocytes, without significant changes in the frequency of the three subsets classified through CD14 and CD16 biomarkers analysis.

### CD80/CD163 balance in circulating monocytes from younger and older healthy subjects

The inflammatory and immunomodulatory profile of CD14/CD16 monocytes was further characterized through the expression of CD80 and CD163 in the three monocyte subsets (Figure 1).

**Figure 3:**
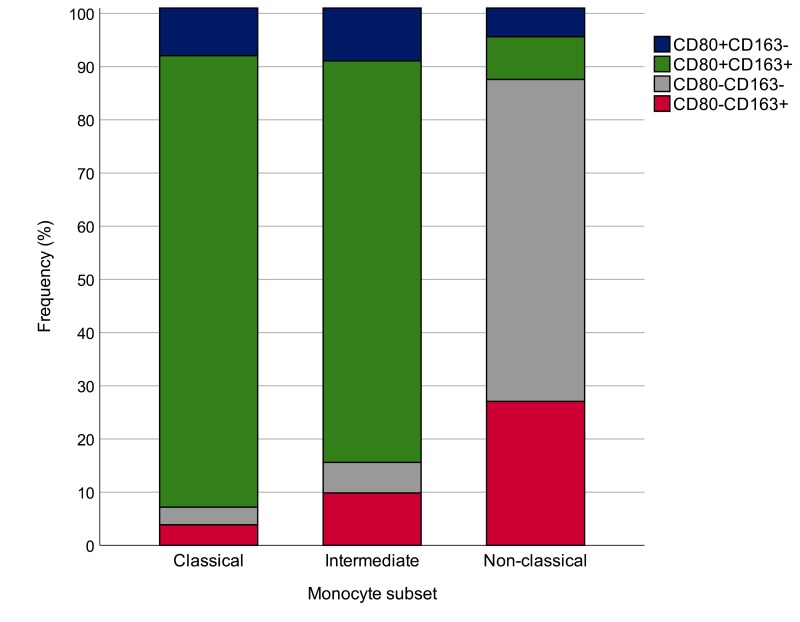
**Proportions of monocytes expressing the four CD80/CD163 biomarker combinations, such as CD80^+^, CD163^+^, CD80^+^CD163^+^ and CD80^-^CD163^-^.** The analysis was performed in 31 healthy subjects. χ^2^ (6) = 169.40, p=0.0001.

Calculation of the proportion of monocytes expressing the four CD80/CD163 biomarker combinations, such as CD80^+^, CD163^+^, CD80^+^CD163^+^ and CD80^-^CD163^-^ in 31 healthy individuals demonstrated that most classical and intermediate monocytes were CD80^+^CD163^+^, whereas non-classical monocytes were most often CD80^-^CD163^-^ and CD163^+^ (χ^2^ (6) = 169.40, p<0.0001) (Figure 3).

**Figure 4:**
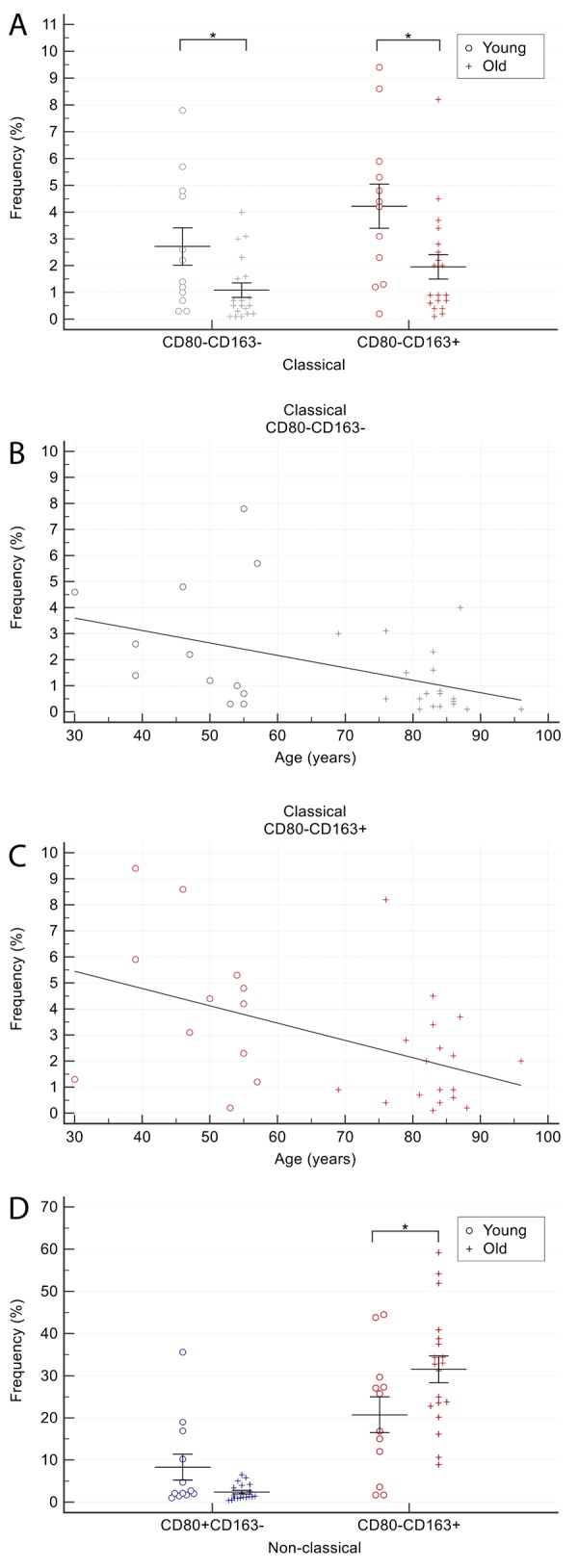
**Comparison of CD80/CD163 cells proportion among monocyte subsets in older and younger CTR subjects.** (**A**) proportion of CD80-CD163- and CD163+ cells among classical monocytes. (**B**-**C**) age-related correlation of CD80-CD163- and CD163+ classical monocytes. (**D**) proportion of CD80+ and CD163+ among non-classical monocytes. Young = 12 healthy subjects younger than 65 years. Old = 19 healthy subjects older than 65 years. Data are expressed as mean±SEM. *p<0.05.

The proportion of CD163^+^ cells among classical monocytes was significantly lower in older than in younger CTR subjects (2.1±2.2 *vs*. 4.2±2.8; p=0.03). The same was true for CD80^-^CD163^-^ cells (1.1±1.2 *vs*. 2.7±2.4; p=0.048) (Figure 4A). The frequency of CD80^-^CD163^-^ (Figure 4B, Spearman’s rho, 0.68; p<0.01) and CD163^+^ (Figure 4C, Spearman’s rho, 0.56; p<0.01) classical monocytes correlated inversely with age.

Intermediate monocytes showed similar features, but differences were not significant (data not shown).

The proportion of CD163^+^ cells among non-classical monocytes was significantly higher in older than in younger CTR subjects (31.1±13.8 *vs*. 20.4±14.9, p<0.05) (Figure 4D). In contrast, the proportion of CD80 single-positive cells tended to be lower in older than in younger CTR individuals (2.4±1.9 *vs*. 8.3±10.6), although the difference was not significant (p=0.08) (Figure 4D).

Absolute numbers of M1 and M2 classical and non-classical monocyte subsets, calculated from the absolute numbers of total monocytes, showed the same trends observed for proportions (data not shown).

Collectively, these data suggest that healthy aging is associated with i) a reduction in CD163^+^ and CD80^-^CD163^-^ classical monocytes and ii) an increase in CD163^+^ non-classical monocytes.

### NK and NK/T cells frequency in healthy subjects

The frequency of NK (CD16^+^CD56^+^CD3^-^) and NK/T (CD16^+^CD56^+^CD3^+^) cells was also calculated. For the purpose of the study, single-positive (CD16 or CD56) and double-positive cells were pooled.

**Figure 5:**
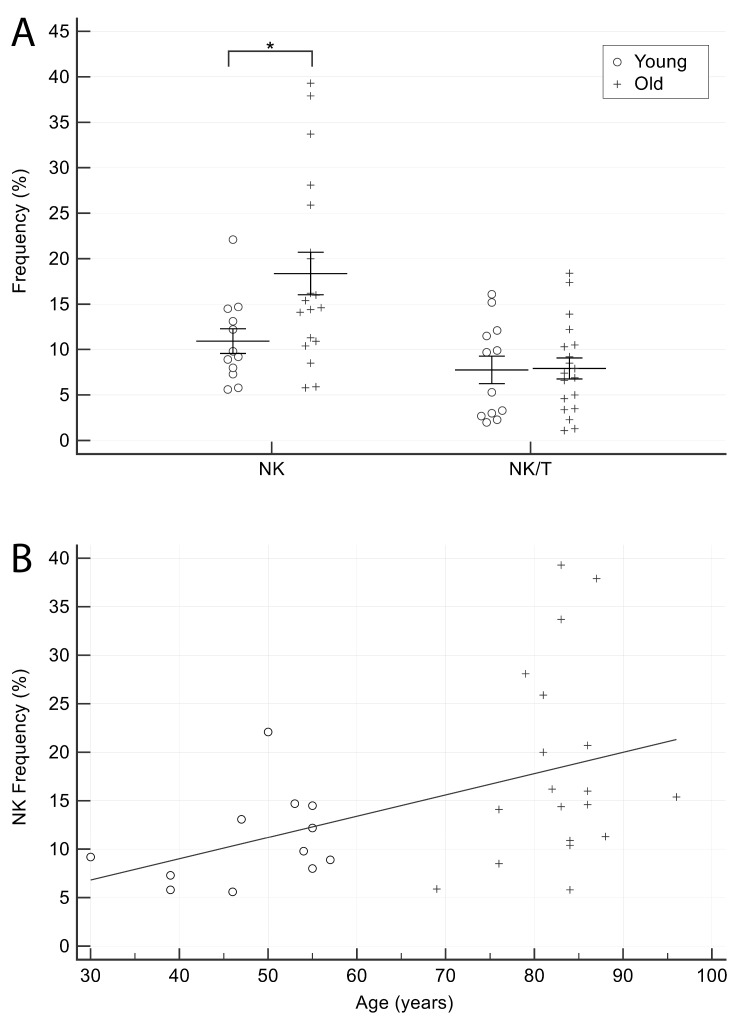
**Comparison of NK cells proportion in older and younger CTR subjects.** (**A**) NK and NK/T cells in younger and older CTR subjects. (**B**) Age-related correlation of NK cells. Young = 12 healthy subjects younger than 65 years. Old = 19 healthy subjects older than 65 years. Data are expressed as mean±SEM. *p<0.05.

The proportion of NK cells was significantly higher in older CTR (Figure 5A), and showed a significant positive correlation with age (Spearman’s rho= 0.44, p=0.01) (Figure 5B), whereas the proportion of NK/T cells did not display significant age-related differences.

### Monocyte frequency in AMI patients

Monocytes – either CD14^+^ cells and those belonging to each of the three subsets – exhibited no significant differences between AMI patients and older CTR subjects (data not shown).

### CD80/CD163 balance in circulating monocytes from AMI patients and older controls

**Figure 6:**
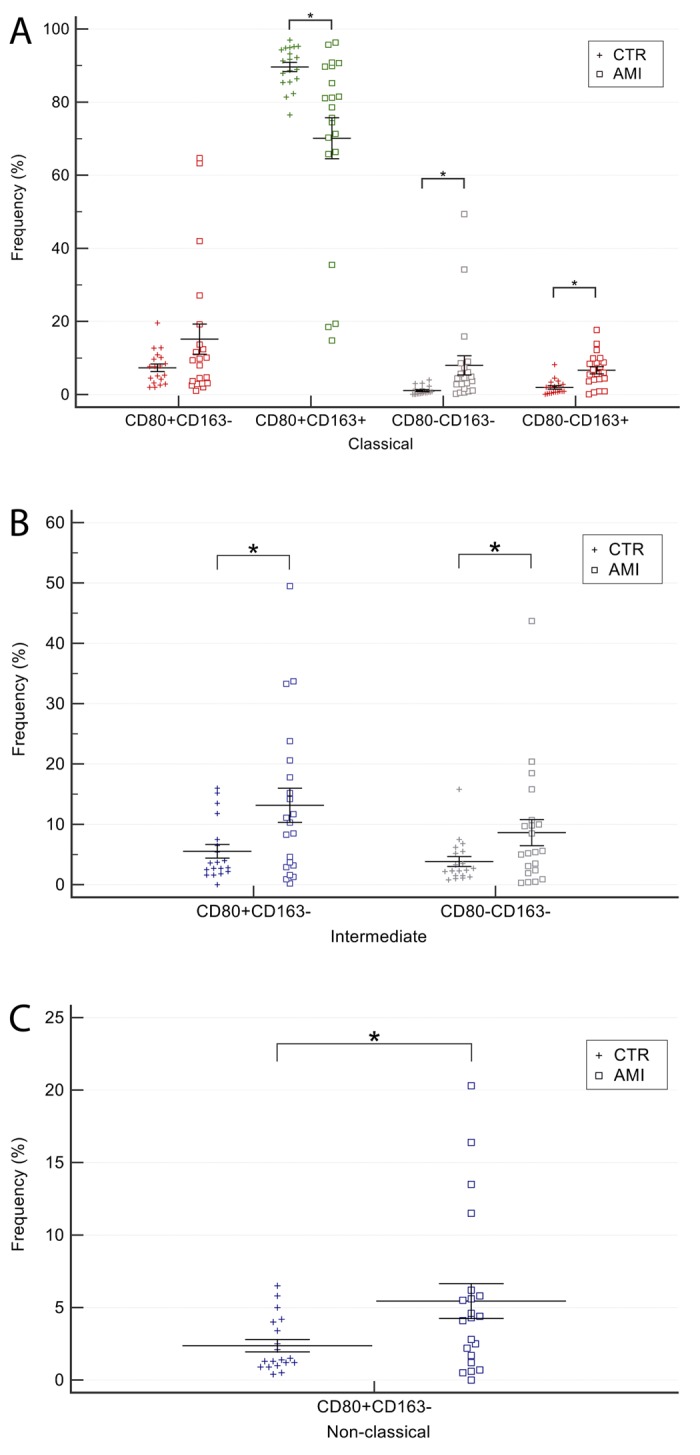
**Comparison of CD80/CD163 monocytes subsets among classical, intermediate and non-classical monocytes in AMI patients and older CTR subjects.** (**A**) Classical, (**B**) intermediate, (**C**) non-classical monocytes. AMI = 21 patients affected by AMI, older than 65 years. Old = 19 healthy subjects older than 65 years. Data are expressed as mean±SEM. *p<0.05.

Comparison of CD80 and CD163 expression in classical, intermediate, and non-classical monocytes from AMI patients and older CTR individuals demonstrated a significantly lower proportion of CD80 and CD163 double-positive classical monocytes in AMI patients compared to older CTRs (70.1±25.7 *vs*. 89±5.6, p<0.01) and a significantly higher proportion of the CD80^-^CD163^+^ (6.7±4.6 *vs.* 2.1±2.2, p<0.01) and CD80^-^CD163^-^ subsets (8.2±12.1 *vs*.1.1±1.2, p=0.02) (Figure 6A).

CD80 single-positive cells were more numerous in AMI patients (15.2±18.9 *vs*. 7.3±4.6), but the difference was not significant (p=0.08).

As regards intermediate monocytes, double-negative cells were significantly more frequent in AMI patients (8.61±9.91 *vs.* 3.81±3.62, p=0.049), as well as CD80 single-positive cells (13.11±12.96 *vs*. 5.52±4.91, p=0.02) (Figure 6B).

Finally, AMI was associated with a greater frequency of non-classical CD80 single-positive cells (5.4±5.5 *vs.* 2.4±1.9, p=0.02) (Figure 6C).

Altogether, these data highlight that AMI patients are characterized i) by extensive and complex changes in monocytes, particularly the classical and intermediate subsets; and ii) by substantial monocyte activation and differentiation, especially a decline of CD80 and CD163 double-positive (quiescent) classical monocytes and an increase in CD163^+^ cells, an opposite trend to that observed in healthy aging. Moreover, the proportion of CD80 single-positive (M1 phenotype) cells increased in intermediate and non-classical monocytes.

**Figure 7:**
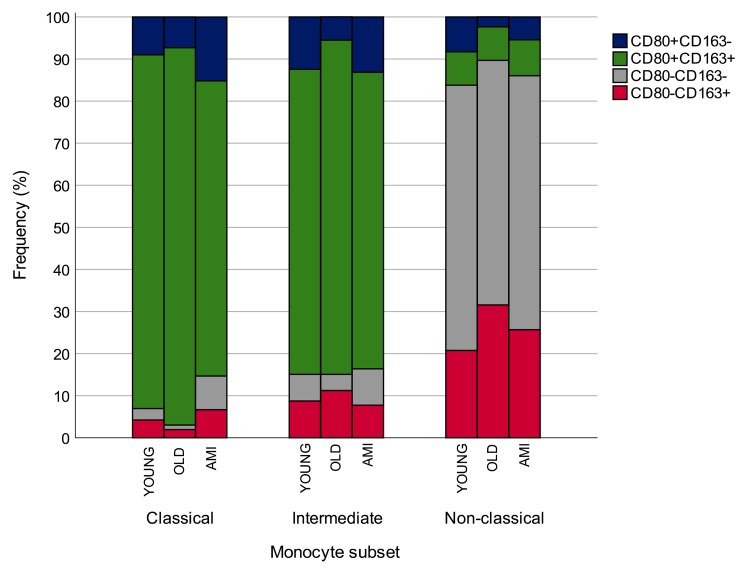
**Percentage of CD80/CD163 expressing cells among classical, intermediate and non-classical circulating monocytes in younger and older CTR subjects and in AMI patients.** AMI = 21 patients affected by AMI, older than 65 years. Old = 19 healthy subjects older than 65 years. *p<0.05.

The percentage of M1/M2 cells among classical, intermediate and non-classical circulating monocytes in younger and older CTR subjects and in AMI patients is summarized in Figure 7.

### NK and NK/T cells frequency in AMI patients

**Figure 8:**
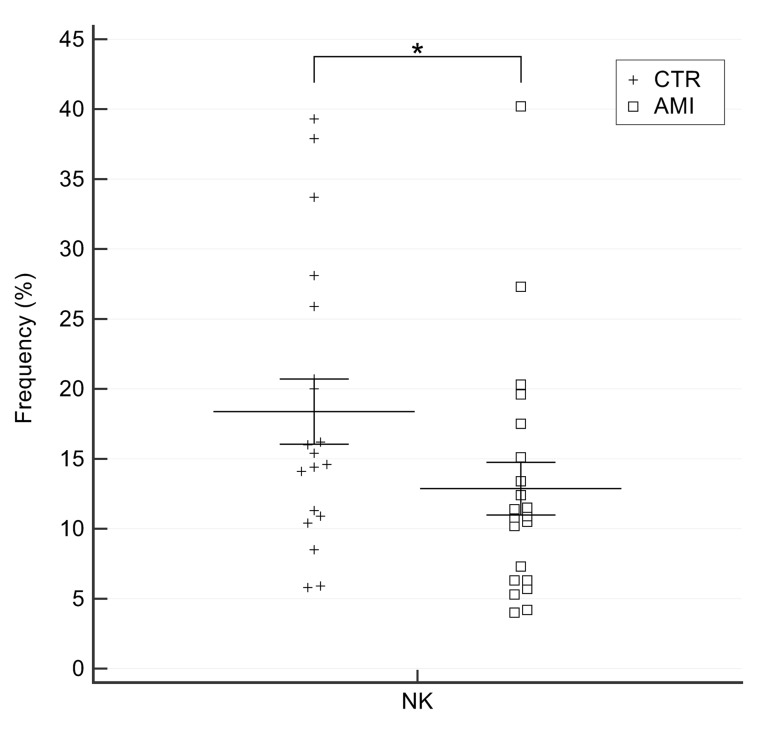
**Percentage of NK cells in AMI patients and old CTR subjects.** AMI = 21 patients affected by AMI, older than 65 years. Old = 19 healthy subjects older than 65 years. Data are expressed as mean±SEM. *p<0.05.

A significantly lower number of NK cells was detected in AMI patients compared with age-matched CTR individuals (11.51±6.08 *vs.* 18.52±10.12, p=0.014) (Figure 8**).** No significant changes were observed in NK/T subset (data not shown).

These data strengthen the hypothesis of a protective role of innate immune cells, particularly NK cells, against ischaemic heart disease.

## DISCUSSION

The present study was conducted to investigate the M1/M2 profiles of circulating monocytes in healthy individuals of different ages. A significantly higher proportion of total monocytes (CD14^+^) was found in the older subjects, whereas no major age-related changes were observed when monocytes were classified into classical, intermediate and non-classical, in relation to CD14 and CD16 expression. The pro- or anti-inflammatory profiles of circulating monocytes were further characterized by CD80 and CD163 expression. Classical and intermediate monocytes consist predominantly of CD80 and CD163 double-positive subpopulations which could be considered as low polarized “resting” reservoirs that can differentiate into pro-inflammatory (CD80) and/or immunomodulatory (CD163) cells in different physiological and pathological conditions. Surprisingly, non-classical monocytes were mainly represented by CD80^-^CD163^-^ and CD163^+^ cells.

A significant age-related increase was observed in CD163^+^ non-classical monocytes, whereas an age-related reduction of CD163^+^ cells was detected among classical monocytes, suggesting different age-related trends for classical and non-classical M2 monocytes. Overall, since classical monocytes account for 80-90% of circulating monocytes, healthy aging processes seem to be characterized by a reduced proportion of M2 monocytes. The reason for this age-related change is unclear. Recent studies of monocyte polarization show that macrophages with an anti-inflammatory phenotype, *i.e.* M2, may produce excessive pro-inflammatory mediators [16], whereas non-classical monocytes are prone to acquire a senescent phenotype characterized by a pro-inflammatory secretome, a condition that is seen in elderly individuals [17]. Notably, non-classical monocytes from our older healthy participants displayed a higher proportion of CD163^+^ cells, suggesting that this feature could be a new piece in the complex puzzle of macrophaging [2]. Moreover, an increased proportion of CD163^+^ monocytes has been described in different ARDs such as type 2 diabetes [12] and cancer [13], lending further support to the hypothesis that healthy aging could be associated with a reduced proportion of M2-monocytes.

To unravel the M1/M2 imbalance in old ARD patients, we analysed a sample of patients who had experienced an acute cardiovascular event (AMI). We found no major changes in the three main monocyte subsets in these patients compared with old CTRs. Although some studies have described a redistribution of circulating monocyte subsets in patients with cardiovascular disease (CVD), their data are inconsistent [18,19]. Problems like monocyte quantification (absolute number or proportion) and the different timing of sample collection may have contributed to these inconclusive results [20,21].

Comparison of old healthy CTRs and AMI patients indicated that in the latter group classical monocytes exhibited a significant reduction of CD80 and CD163 double-positive (quiescent) cells and a significant increase in CD163^+^ monocytes, an opposite trend to that observed in healthy aging. These findings agree with studies reporting that CD163^+^ macrophages have a role in promoting atherosclerosis by enhancing inflammation and vascular permeability [22], and that they contribute to cardiac remodelling after AMI (reviewed by Hulsmans [23]). Recently, soluble CD163 has emerged as an innovative marker of inflammation and macrophage activation in a number of pathological conditions [24–27] including CVD [28].

In addition, the proportion of CD80 single-positive (M1 phenotype) cells increased significantly in intermediate and non-classical monocytes, supporting the hypothesis of an additional pro-inflammatory polarization of monocytes in an acute cardiovascular event.

Finally, we observed an age-related increase in the frequency of NK cells – which supports the notion that these cells play a role in promoting healthy aging, probably through the clearance of senescent cells [29]) – as well as of cancer cells [30]. In contrast, their proportion was significantly lower in AMI patients, in line with studies showing that poor NK function is associated with increased morbidity and mortality [31–35].

In conclusion, cytofluorimetric analysis proved to be a fast and practical approach to study M1/M2 polarization including CD80 and CD163 evaluation besides the biomarkers CD14 and CD16. Further work is required to confirm these preliminary findings and clarify the functional significance of age-related monocyte phenotype changes.

## MATERIALS AND METHODS

### Subjects

Thirty-one healthy subjects and 21 AMI patients were enrolled at the Ancona National Institute for Health Care of the Elderly (INRCA) in the framework of an Italian national study directed at identifying the biological parameters associated with healthy/unhealthy aging. The study protocol was approved by the INRCA ethics board. Participants signed an informed consent before enrolment.

Healthy subjects were further divided in two subgroups, younger (n=12) and older (n=19), according to age (<65 years and ≥65 years), in order to obtain two study groups that were statistically homogeneous and comparable.

None of the participants had a previous history of cancer. All subjects (CTR and AMI) underwent complete physical examination, as well as haematological, biochemical and instrumental examination to rule out presence of any co-existing pathology. Detailed medical history was collected from all subjects, with particular attention paid to previous and current diseases, use of medications and smoking habits. Ex smokers who had given up smoking for a period of at least 3 years were considered as non-smokers. None of the healthy controls reported the chronic use of any medication. Moreover, healthy subjects were advised to avoid occasional intake of any drug or supplement during the 7 days preceding collection of blood samples.

AMI was diagnosed according to European Society of Cardiology (ESC) guidelines (available at https://www.escardio.org). Samples from AMI patients were collected in the first week after the cardiovascular event, but time course data were not available. AMI patients received standard treatment according to current guidelines [36].

Characteristics of study population are summarized in Table 1.

### Laboratory assays

White blood cell (WBC) count, haemoglobin (Hb), glucose, total cholesterol, and hs-TnT were determined by standard automated procedures.

### Flow cytometry

Peripheral venous blood was collected into EDTA-coated tubes (Venoject, Terumo Europe NV, Leuven, Belgium). Whole blood samples were placed into EDTA-containing tubes. Six-colour flow cytometric analysis was performed with FacsCanto II (Becton-Dickinson, Franklin Lakes, NJ) after labelling 100 μl of blood with the following conjugated monoclonal antibodies: CD14* allophycocyanin (APC), CD16* fluorescein isothiocyanate (FITC), CD80* phycoerythrin-cyanine 7 (PE-Cy7), CD163* phycoerythrin (PE), CD3* Allophycocyanin (APC) and CD56* PE (all from Becton-Dickinson‎). Cells were incubated for 30 min at room temperature with the optimal dilution of each antibody, according to the manufacturer’s instructions. The frequencies of the different subpopulations were calculated using FacsDiva software (Becton-Dickinson). Monocytes and natural killer (NK) cells were processed in separate tubes. At least 200,000 (monocyte typing) and 50,000 events (NK cells typing) were acquired.

### Statistical analysis

Comparisons among groups were performed with univariate or multivariate analysis of variance (respectively ANOVA and MANOVA), as appropriate. Correlations between parameters were calculated using Spearman’s rho. Chi square test was used as appropriate. Data were analysed with the SPSS/Win program version 22 (SPSS, Chicago, IL). Statistical significance was defined as a two-tailed p value < 0.05.
